# Microevolutionary analysis of *Clostridium difficile *genomes to investigate transmission

**DOI:** 10.1186/gb-2012-13-12-r118

**Published:** 2012-12-21

**Authors:** Xavier Didelot, David W Eyre, Madeleine Cule, Camilla LC Ip, M Azim Ansari, David Griffiths, Alison Vaughan, Lily O'Connor, Tanya Golubchik, Elizabeth M Batty, Paolo Piazza, Daniel J Wilson, Rory Bowden, Peter J Donnelly, Kate E Dingle, Mark Wilcox, A Sarah Walker, Derrick W Crook, Tim E A Peto, Rosalind M Harding

**Affiliations:** 1 Department of Statistics, University of Oxford, 1 South Parks Road, Oxford OX1 3TG, UK; 2 Nuffield Department of Clinical Medicine, University of Oxford, John Radcliffe Hospital, Headley Way, Oxford, OX3 9DU, UK; 3 Oxford Biomedical Research Centre, John Radcliffe Hospital, Headley Way, Oxford OX3 9DU, UK; 4 Wellcome Trust Centre for Human Genetics, Roosevelt Drive, Oxford OX3 7BN, UK; 5 Nuffield Department of Clinical Laboratory Sciences, Headley Way, University of Oxford, John Radcliffe Hospital, Oxford, OX3 9DU, UK; 6 Department of Microbiology, The General Infirmary, Old Medical School, Great George Street, Leeds LS1 3EX, UK; 7 Leeds Institute of Molecular Medicine, University of Leeds, Beckett Street, Leeds LS9 7TF, UK; 8 MRC Clinical Trials Unit, 125 Kingsway, London, WC2B 6NH, UK; 9 Department of Zoology, University of Oxford, South Parks Road, Oxford OX1 3PS, UK

## Abstract

**Background:**

The control of *Clostridium difficile *infection is a major international healthcare priority, hindered by a limited understanding of transmission epidemiology for these bacteria. However, transmission studies of bacterial pathogens are rapidly being transformed by the advent of next generation sequencing.

**Results:**

Here we sequence whole *C. difficile *genomes from 486 cases arising over four years in Oxfordshire. We show that we can estimate the times back to common ancestors of bacterial lineages with sufficient resolution to distinguish whether direct transmission is plausible or not. Time depths were inferred using a within-host evolutionary rate that we estimated at 1.4 mutations per genome per year based on serially isolated genomes. The subset of plausible transmissions was found to be highly associated with pairs of patients sharing time and space in hospital. Conversely, the large majority of pairs of genomes matched by conventional typing and isolated from patients within a month of each other were too distantly related to be direct transmissions.

**Conclusions:**

Our results confirm that nosocomial transmission between symptomatic *C. difficile *cases contributes far less to current rates of infection than has been widely assumed, which clarifies the importance of future research into other transmission routes, such as from asymptomatic carriers. With the costs of DNA sequencing rapidly falling and its use becoming more and more widespread, genomics will revolutionize our understanding of the transmission of bacterial pathogens.

## Background

*Clostridium difficile *infection (CDI) has been a substantial burden on healthcare facilities over the past decade [1-3]. A widely held assumption that much transmission occurs in hospitals between symptomatic patients was reinforced when enhanced infection control introduced in England in 2007 was followed by declines in the incidence of CDI [3]. Identifying routes of nosocomial transmission for lineages of *C. difficile *is an important step towards further improvements of infection control. Clinical isolates of *C. difficile *have been typed using a wide variety of methods [4], but these schemes on their own are not sufficiently discriminatory to investigate propagation patterns on a fine scale. For example, a highly pathogenic *C. difficile *lineage emerged clinically 10 years ago [5,6] and has until recently been responsible for up to 40% of *C. difficile *infections reported in the United Kingdom [7,8]. Isolates from this lineage are undistinguishable by conventional typing methods, since they all correspond to a single PCR ribotype denoted 027 [3] and a single multi-locus sequence typing (MLST) [9,10] type denoted ST1 [8,11].

In a previous study [12], we used comprehensive epidemiological information on patient admissions and ward movements within the Oxfordshire hospitals [13] to discriminate routes of nosocomial transmission between symptomatic cases sharing the same MLST type. This study found fewer cases of CDI than anticipated that could be attributed to acquisitions from other symptomatic patients sharing space and time on a hospital ward [12]. One difficulty for the wider application of this epidemiological approach is that the availability of high-quality patient records is unusual. More typically, omissions or inaccuracies in such databases reduce successful record linkage [13]. Furthermore, patient pathways are only indirectly informative about transmission, in the sense that contact between patients does not imply transmission, and conversely, transmission can take place during unrecorded chance encounters in hospital facilities or via third parties who were not sufficiently ill to be sampled. We therefore sought a new strategy for transmission analysis that does not require epidemiological data. Instead, whole genomes were sequenced to provide a genetic resolution that is directly informative about fine scale patterns of transmission.

Comparisons of whole genome sequences have already led to new insights into the epidemiology of other bacterial pathogens such as *Staphylococcus aureus *[14] and *Streptococcus pneumoniae *[15]. The sequencing of the first whole genome of *C. difficile *revealed that it contains many mobile genetic elements, which could contribute to its pathogenicity and evolving antibiotic resistance [16]. A comparison of a limited number of genomes (n = 30) showed that the current diversity of *C. difficile *is the result of a complex evolutionary history involving frequent horizontal gene transfer and homologous recombination [17]. An important development in infectious disease research, termed 'phylodynamics', aims to improve our understanding of the relationships between the genetic variation of pathogens and their epidemiology [18]. Although applicable in principle to all kinds of pathogens, the majority of these studies to date have focused on viral infectious diseases [19]. However, the development of next generation sequencing technologies [20] has made the unification of epidemiological and evolutionary approaches feasible for bacterial pathogens as well.

For this study we sequenced, with high quality, genomes from 486 CDI cases arising in Oxfordshire between September 2006 and June 2010. These included a third of the 1,460 cases reported during this period, and those through to December 2009 have been previously studied by MLST [8]. The Oxford University Hospitals (OUH) provide all acute services for the region, and are therefore, *a priori*, considered the most likely place of transmission. To test this hypothesis, we reconstructed precise genealogies relating the *C. difficile *isolates, and used these to assess the plausibility of transmission between cases. We then compared the results of our genomic analysis of plausible transmission events with the proposed patient links that were determined from hospital admissions and ward movements over the same period [12].

## Results

### Whole genome sequencing, quality control and molecular clock

All genomes in this study were sequenced using Illumina technology [21], with reference-based assemblies performed using STAMPY [22] against the genome sequence of strain 630 [16]. Additional variant calling filters were applied to maximize the quality of the genome sequences generated (Materials and methods). We performed the sequencing, assembly and calling of 66 samples between 2 and 8 times in order to assess robustness, providing a total of 189 sequences (30 samples sequenced twice, 28 samples 3 times, 4 samples 5 times, and 1 sample 4, 6, 7 and 8 times). Across 224 comparisons, 222 pairs of sequences were identical, and the remaining two pairs differed by only a single nucleotide. Thus, we estimate that our error rate is of the order of one mistake every 100 genomes sequenced. If the probability of one error arising in a genome is approximately 1%, and assuming that errors happen independently (and the results above show no indication that they do not), then the probability of two errors is approximately 0.01%. This low error rate gives us confidence that small numbers of pairwise differences between genomes accurately reflect mutational divergence.

In order to estimate the molecular clock, serial pairs of isolates sampled from 91 CDI cases and sharing the same ST, but separated by 1 to 561 days, were whole-genome sequenced (Additional file 1). The within-host evolutionary rate of *C. difficile *was estimated at µ = 3.2 × 10^-7 ^mutations per site per year, with a 95% credibility interval ranging from 1.3 × 10^-7 ^to 5.3 × 10^-7^. Our calculation accounted for the instantaneous diversity of *C. difficile *within a host, and this quantity was as observed in experiments where several genomes were sequenced from the same host and timepoint (Materials and methods). Our estimate of the short-term molecular clock rate is two orders of magnitude larger than a previous long-term molecular clock estimate [17]. Similar discrepancies between short-term and long-term rates have been reported in other pathogens - for example, in *Campylobacter jejuni *[23] or *Helicobacter pylori *[24] - and could be due to several evolutionary factors [25]. This result highlights the importance of using a short-term molecular clock to date recent evolutionary events [24-26].

Our short-term molecular clock rate represents an average of 1.4 mutations per genome per year (with a 95% credibility interval ranging from 0.6 to 2.3), and values of the same order of magnitude have been recently reported from whole genome comparisons of various bacterial species [27], including *Mycobacterium tuberculosis *[28], *Vibrio cholerae *[29], *Escherichia coli *[30] and *Staphylococcus aureus *[31]. Note that although these bacterial rates of evolution per site are lower than in viruses, the longer length of bacterial genomes means that rates of genomic evolution are comparable. For example, the hepatitis C virus, which has a genome of 9,600 bp, accumulates substitutions at a rate of 0.79 × 10^-3 ^per site per year [32], equivalent to 7.6 substitutions per genome per year. This genomic rate is of the same order of magnitude as the rate reported here for *C. difficile*.

### Concordance with MLST

**Figure 1:**
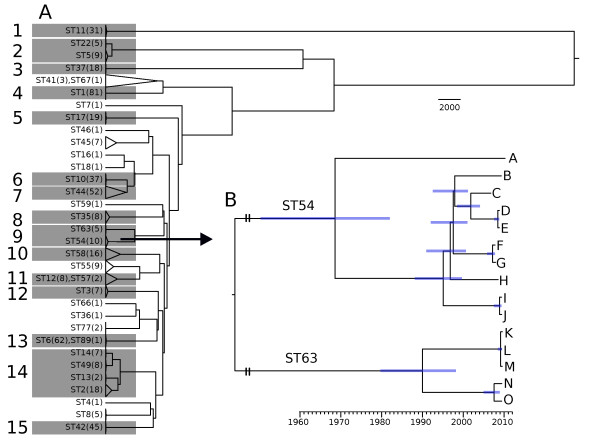
**Global and group-by-group phylogenetic analysis of the genomes**. **(a) **Phylogeny of all 486 genomes from included CDI cases. Clades corresponding to STs are shown as triangles, the height of which represents the diversity of each ST. Numbers in parentheses indicate the number of genomes belonging to each ST sequenced. The 15 STs or groups of closely related STs analyzed in detail are shaded, and indexed from 1 to 15. **(b) **Detailed phylogeny for samples in group 9. The ten genomes of ST54 are labeled from A to J and the five genomes of ST63 are labeled from K to O. The × co-ordinate of each genome indicates time of isolation. For each internal node of the tree (corresponding to a common ancestor), the mean inferred age is shown on the tree with the 95% credibility interval around this mean shown as a blue bar.

Clustering of single genomes from each of the 486 CDI cases (Additional file 2) by UPGMA (Figure 1a) confirmed the correspondence of major phylogenetic lineages to STs. Three exceptions, ST89, ST57 and ST67, fell within the diversity of ST6, ST12 and ST41, respectively. Such inclusions can be easily explained; for example, for ST89 by clonal expansion of a genetically differentiated descendant of the ST6 lineage. Provided that MLST data are not wrongly interpreted as implying the monophyly of each ST, such exceptions do not represent inconsistencies between MLST and whole-genome phylogeny. A previously described example is the inclusion in *Neisseria meningitidis *of ST66 within the diversity of ST8 [33].

The extent of genomic diversity within lineages was highly variable (indicated by the height of the triangles in Figure 1a) between STs. In these data, ST44 presents the two most divergent genomes that share the same sequence type, with one pair differing at 4,273 sites. The minimum number of differences between two genomes belonging to separate STs (excluding the nested STs above) was 771, representing over a hundred years of evolution. Grouping by MLST is therefore consistent with the phylogenetic structure evident from whole genome data, but finer resolution is necessary to study epidemiological events.

### Extent of recombination

In the light of strong evidence for extensive homologous recombination in *C. difficile *[8,17,34], the microevolutionary history of 15 clades represented in our data (Figure 1a) was reconstructed independently using ClonalFrame [35]. Estimates of the relative effects of mutation and recombination (r/m) [34,36] varied by two orders of magnitude between lineages (Table 1), with values above 5 in groups 1, 11 and 14 and values below 0.05 in groups 3 and 4. The relative effect of recombination has previously been estimated to be between 0.1 and 0.4 in *C. difficile *based on MLST data [34,36], but recombination is likely to play a lesser role on the highly conserved MLST loci than it does throughout the genome [8,36]. A comparison of whole genomes covering the complete diversity of *C. difficile *yielded an estimate between 0.63 and 1.13 [8,34], which falls roughly at the middle of the interval of values estimated on a group-by-group basis (Table 1).

**Table 1:** Properties of the 15 groups highlighted in Figure 1a

Group	STs	Counts	min(d)^a^	max(d)^a^	mean(d)^a^	Mut^b^	Rec^c^	Sub^d^	r/m^e^
1	11	31	0	285	46	166	61	865	5.22
2	5,22	9,5	0	1,202	614	364	49	1,364	3.75
3	37	18	0	49	22	100	1	3	0.03
4	1	81	0	24	6	79	1	3	0.04
5	17	19	0	114	42	161	18	191	1.19
6	10	37	0	120	30	273	22	155	0.57
7	44	52	0	4,273	911	2,226	135	4,134	1.86
8	35	8	0	1,026	307	621	9	661	1.07
9	54,63	10,5	0	6,009	2,633	2,238	180	5,678	2.54
10	58	16	0	3,130	1,306	880	149	3,845	4.37
11	12,57	8,2	0	2,953	1,368	426	78	3,189	7.49
12	3	7	2	468	225	386	21	454	1.18
13	6,89	62,1	0	344	84	1,197	255	1,784	1.49
14	2,13,49,14	18,2,8,7	0	3,913	1,675	913	201	6,495	7.11
15	42	45	0	155	24	206	22	178	0.87

^a^d is the pairwise distance between two genomes from different CDI cases. ^b^Estimated number of mutation events. ^c^Estimated number of recombination events. ^d^Estimated number of substitutions introduced by recombination. ^e^Ratio of the effects of recombination versus mutation.

Our results show that the effect of recombination varies substantially between lineages of *C. difficile*. Evidence for non-constant effects of recombination have been reported previously in other pathogenic species such as *Moraxella catarrhalis *[37], *Listeria monocytogenes *[38] and *Escherichia coli *[39]. These variations are often found associated with a change in ecology or pathogenicity [36]. ST1, the hypervirulent strain associated with major hospital outbreaks and severity over the last decade [5,6], displayed very little evidence for recombination, and therefore may be best described as a monomorphic pathogenic clone [40]. Interestingly, ST37 (PCR-ribotype 017), the only toxin A-B+ strain in this study [8], shared this feature.

### Epidemiological interpretation of genealogies

The microevolutionary history for group 9 chosen to illustrate epidemiological interpretation is shown in Figure 1b (equivalent genealogies for the other 14 groups are shown in Additional file 3). Once the recombination events inferred by ClonalFrame are accounted for, time-scaling is informed by the molecular clock estimate µ and the known dates of isolation of each genome. Patients K to O represent all five cases of ST63 arising during the 3.5 year study period, which actually occurred over an interval of 9 weeks. Although one of these samples was obtained by a family doctor in primary care and the others were taken in the three separate OUH hospitals, K, L, and M had actually shared time on one ward, while N and O shared time on another. Our independent genomic analyses revealed that the estimated date of the common ancestor of K, L and M could be as little as a few days before samples were taken (these three genomes have no observed differences), consistent with transmission on the ward between these three patients. Thus, epidemiology confirms the genetic signal of possible nosocomial transmission. Likewise, we cannot exclude the possibility of direct transmission between N and O, given that the time back to their most recent common ancestor (TMRCA) may be as short as five months. However, any direct transmission between K, L, and M on the one hand, and N and O on the other, can be ruled out on the basis that their TMRCA is at least ten years before the first isolation. All five patients had received treatment from clinicians in the same speciality, raising concerns that they were spreading CDI between hospitals, but our whole genome sequencing provides strong evidence against this hypothesis. During the study period there were nine isolations of ST54, within which three pairs of genomes were closely related (D and E, I and J, F and G). Only D and E shared time on the same ward. I and J were on adjacent wards at the same time, whereas F and G were never in the same OUH hospital at the same time nor in the same part of the ward or even hospital area at different times, suggesting either transmission outside of the OUH, or the involvement of a third party (either an asymptomatic carrier or a false-negative tested patient). Strains isolated from four other patients (A, B, C and H) had no close relatives (Figure 1b).

### Assessing the plausibility of direct transmission between two cases

**Figure 2:**
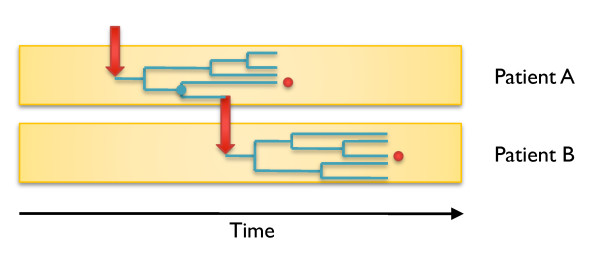
**Conceptual representation of how the TMRCA relates to transmission**. Two patients A and B are shown, and a transmission event happened from A to B as indicated by a red arrow. Within each patient, a colonizing population evolves between the time of infection and the time when the patient becomes symptomatic, at which point a single strain is isolated, represented by a red dot. The most recent common ancestor of the two isolates from A and B is shown as a blue dot.

In order to interpret the dated genealogies reconstructed by ClonalFrame (Figure 1b; Additional file 3) for all 486 cases, let us consider what happens when transmission occurs between two patients (Figure 2). It is important to notice that the strains isolated and sequenced from each of the two patients (represented by red dots in Figure 2) only represent single members of the bacterial populations colonizing these hosts. Nevertheless, the TMRCA of the two sampled genomes (represented by a blue dot in Figure 2) can be estimated and necessarily predates the transmission event [41]. Let the incubation period be defined as the time separating the infection (red arrow) from the symptomatic phase during which a sample is taken (red dot). For nosocomial CDI this is estimated to be days to weeks rather than months [42]. The directionality of a putative transmission event is not generally known, but it is clear from Figure 2 that if transmission occurred in one or other direction, the TMRCA is at most two incubation periods prior to either sample being taken.

**Figure 3:**
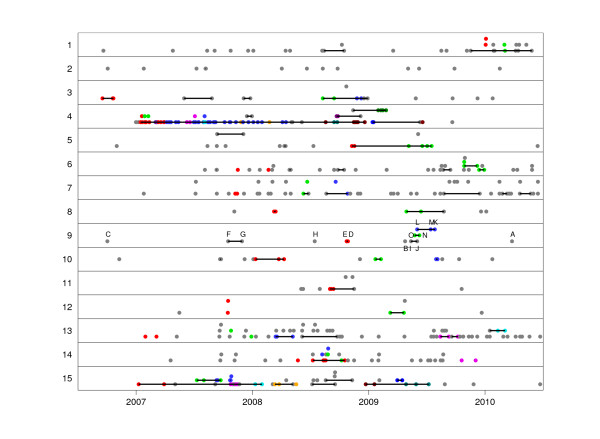
**Summary of results of the transmission analysis**. Each horizontal panel corresponds to one of the 15 groups defined in Figure 1a. Each circle represents a CDI case, and is located on the x-axis according to the isolation date. Cases are linked by horizontal lines where the genomic analysis found that transmission was possible. The 15 genomes in group 9 are labeled from A to O as in Figure 1b. Within each group, cases that are connected with one another based on the epidemiological analysis are indicated by circles of the same unique color, and cases with no identified epidemiological connections are shown in gray. The same colors are used to label isolates in Additional file 3.

**Figure 4:**
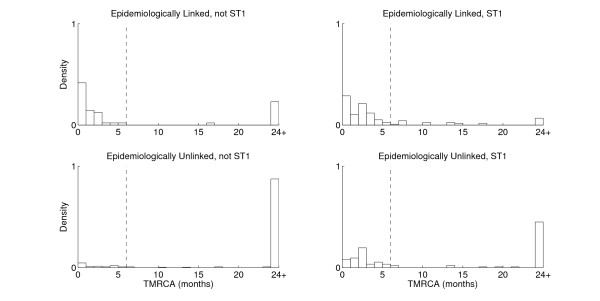
**Distributions of the TMRCA for all pairs of genomes with the same ST and isolated within a month of each other**. Top left: 48 pairs not from ST1 and with an epidemiological link based on shared time and space on a ward. Bottom left: 118 pairs not from ST1 and without an epidemiological link. Top right: 310 pairs from ST1 and with an epidemiological link. Bottom right: 149 pairs from ST1 and without an epidemiological link.

The principle described above was applied to all pairs of CDI cases (Figure 3). Putative transmission links within pairs were ruled out if their TMRCA was more than six months. Conversely, if the TMRCA of a pair could be less than six months, transmission between the patients was inferred to be a possibility but not a certainty. The choice of this cut-off at six months is arbitrarily chosen to be conservative in the proportion of direct transmissions ruled out. We note that halving it or doubling it does not result in many changes in interpretation (Figure 4).

### Results of the transmission analysis

We considered all pairs of genomes having the same ST and isolated within a month of each other, so that without whole genome sequencing, transmission would seem likely. With the exception of ST1, only 19% (67/358) of pairs could share a common ancestor within six months (Additional file 4). This proportion was much higher in ST1 (63%, 167/267), consistent with the epidemic nature of this *C. difficile *lineage [5,6]. ST6, in contrast to ST1, showed a very low proportion of pairs for which direct transmission was possible (5%, 5/103). In spite of being a frequent cause of CDI, the epidemiological behaviour of ST6 must differ from that associated with ST1. Similar results were obtained when considering all pairs of genomes isolated within three months of each other (Additional file 4).

The analysis above assumed that evolution takes place at the constant molecular rate µ estimated within patients. However, *C. difficile *forms resistant spores that can survive either within a patient or in the environment for months or even years [2], during which evolution is effectively suspended. Taking this effect into account in a phylogenetic framework would be difficult, but evolutionary interruptions would result in underestimates of the true times to common ancestors, assuming a constant molecular clock. Pairs of cases for which we ruled out transmission would therefore remain unlinked.

### Validation with epidemiological data

In a previous study, we used information on patient ward movements in the OUH to identify which CDI cases had shared hospital time and space [12]. This group of hospitals provide all acute care, and >90% of specialist services within the region. These data were not restricted to the time and place of the sample tested for CDI, to allow for transmission during incubation and pre-symptomatic infection phases. Patients with the same ST that were found to be epidemiologically linked are represented using the same colors on each horizontal panel of Figure 3. Of the 234 pairs of same-ST CDI cases occurring within a month of each other and with a recent (<6 months) common ancestor, 130 (56%) had previously shared time and space in hospital, compared to only 9% (36/391) of the pairs with less recent common ancestors (Figure 4). This association between the genomic and epidemiological analyses is strong and significant (Kappa = 0.49, *P *< 10^-5^; Additional file 5), providing external validation for both.

However, 25% of same-ST pairs from patients sharing time and space on wards had TMRCAs of more than 6 months (Figure 4), thus ruling out direct patient to patient transmission. Again, ST1 stands out compared to other groups, with a higher proportion of epidemiologically unlinked pairs having close genealogical relationships of less than six months (Figure 4). We do not infer that all these pairs represent direct transmissions, because showing this would require the ability to rule in when transmission happened whereas this study focused on ruling out when it did not. Nevertheless, ST1 illustrates the ongoing challenges posed by epidemic outbreaks for identifying transmission pathways, even when genomic and epidemiological data are combined.

## Discussion

This study has identified likely instances of direct transmission that link a subset of 486 cases of CDI arising in Oxfordshire over 4 years. To do so, we first estimated the molecular clock rate of *C. difficile *from genomes sampled serially from within patients, and then applied this rate to reconstruct time-scaled genealogies that can be interpreted epidemiologically. This general phylodynamic approach has been previously applied in several recent viral studies [43-45], but here we used ClonalFrame [35] rather than BEAST [46]. Both algorithms are built on the same concepts of Bayesian phylogenetics [47,48], the main difference being that ClonalFrame accounts for the recombination events, which can disrupt a phylogenetic reconstruction if ignored [49]. Inclusion of variable sites introduced by recombination with those introduced by mutation would result in overestimation of the dates of common ancestors. However, having identified the recombination events, the ClonalFrame algorithm permits TMRCA estimations based on the mutation events only. In line with previous studies [8,17,34], our results indicate levels of recombination in *C. difficile *that are too high to be ignored and justify our approach.

Since our estimated molecular clock is based on short-term data, it may not be suited to date ancient events [24-26]. We therefore interpret with caution the dating of ancient nodes of our phylogenetic trees - for example, the estimated age for the common ancestor of all ST44 of between 400 and 800 years ago (Additional file 3). However, this short-term molecular clock is well suited to estimate the dates of recent events, and in particular to determine whether a pair may have had a common ancestor less than six months ago as required for our epidemiological interpretation.

Only whole-genome sequencing could provide the fine resolution of microevolutionary reconstruction necessary to investigate transmission against a nosocomial background in our study. However, limitations to the conclusions we can draw arise from our sampling design. In particular, we can comment neither on the diversity of the colonizing population within patients nor on the frequency of mixed infections [50], but we can clarify our probabilistic genealogical approach with respect to these concerns. Within-patient diversity is illustrated in Figure 2 where transmission from patient A to patient B is shown. For each patient, there is clonal expansion of the colonizing founder following initial infection (represented by different genealogies within each host). We estimate the time back to the most recent common ancestor (represented by the blue dot) of single isolates sampled from each patient (represented by the two red dots). Irrespective of other diversity in the populations that have colonized patients A and B, the date of this most recent common ancestor (blue dot) has to be after the time of infection of A (first red arrow) and before transmission leads to infection of B (second red arrow). The model of transmission shown in Figure 2 makes the simplifying assumption that a single genome is the founder of each new infection. In reality, each infection may be started by a number of cells or spores, which represents a subsample of the genomic diversity of the donor. However, our estimates of the instantaneous within-host diversity p in *C. difficile *from both longitudinal and synchronous data (Materials and methods; Additional file 6) indicate that this quantity is small, justifying the use of our model.

We have been able to rule out direct transmission for the majority of pairs of patients where it would have seemed likely based on conventional typing methods. For these pairs, our results suggest there must have been a number of intermediates in the transmission chains - or considerably longer incubation periods than have been previously estimated, which seems unlikely for most symptomatic inpatients. Some of these intermediates may be found amongst the reported CDI cases that were not included in our study (486 were sequenced out of 1,460). However, the deep TMRCAs between many of the CDI cases (Figure 4) would require many more intermediates than could be accounted for by the CDI cases not included for genomic sequencing.

The effectiveness of the increased infection control measures initiated in England in 2007 is compatible with the current evidence that transmission between symptomatic hospital inpatients accounts for only a minority proportion of nosocomial CDI acquisitions. The deep TMRCAs between the genomes sampled from many of the epidemiologically linked CDI cases are in line with a growing awareness of community-associated CDI, where conventional risk factors for disease are frequently absent [2,51]. Results presented here therefore highlight the need for future studies to explore transmission routes other than those due to symptomatic inpatient CDI cases. Asymptomatic colonization, albeit potentially transient, may have become an important source of *C. difficile *[52], as indicated by high levels of the general population having serum and colonic antibodies to *C. difficile *toxins [53,54]. Perhaps more importantly, the participation of asymptomatic carriers in chains of transmission could explain the deep TMRCAs found for many pairs of cases (Figure 4). Whilst up to approximately 10% of hospital inpatients have been identified as colonized in prospective studies [55], given the large inpatient pool, asymptomatic colonization could form a substantial reservoir for CDI. Furthermore, the estimates available for *C. difficile *colonization are dependent on bacterial culture, which may underestimate true bacterial prevalence. Current strategies focused on symptomatic patients for reducing nosocomial transmission [56] may therefore have only limited effectiveness, especially as epidemic strains are successfully contained.

## Conclusions

This study demonstrates the utility of microevolutionary analysis based on whole-genome sequencing for studying *C. difficile *transmission. With genomics having an increasing role in the rapid identification of outbreaks, it is clear that microevolutionary analysis of such data will be important to improve clinical management of this endemic healthcare-associated infection. Our methodology applied to future genomic analyses could produce evidence of transmission events as preliminary justification for more detailed epidemiological investigation surrounding particular CDI cases. Analogous genomic studies are likely to illuminate the transmission behaviour and underlying epidemiology of other bacterial pathogens where similarly high rates of genomic microevolution have been found [28-31]. For *C. difficile*, as for many bacterial pathogens, the advent of rapid and affordable sequencing technology [20], and in particular novel rapid benchtop sequencing methods [57], should radically alter our capacity for understanding transmission biology at individual, local and national levels.

## Materials and methods

### Sample collection

A collection of 1,290 clinical isolates of *C. difficile *submitted for testing at the Oxford University Hospitals (Oxford, UK) between September 2006 and December 2009 was previously described [8] and typed using a MLST scheme [11]. This collection has since been extended to include the period from January 2010 to June 2010. It consists of all enzyme immunoassay positive, culture positive isolates from samples submitted to the OUH microbiology laboratory, including from admitted inpatients at other smaller Oxfordshire hospitals (mental health, orthopaedic and community hospitals) and primary care facilities. This laboratory conducts all *C. difficile *testing for Oxfordshire. A subset of this extended collection was studied here: (i) samples from 486 CDI cases (unique patient/ST combination) in 462 different patients, representing 33% of the 1,460 CDI cases during the study period (Additional file 2); (ii) consecutive samples with the same ST isolated from 91 of these CDI cases with time intervals ranging from 1 to 561 days (Additional file 1). Samples for (i) were chosen to represent the breadth of diversity within common disease-causing strains circulating around the OUH at this time (by sequencing at least one sample of all STs with >12 cases in the study period), enhanced for potential transmission events by sequencing as many samples as possible for four wards with ongoing outbreaks as defined by UK Department of Health guidance (three or more cases on a ward in one week) and from STs where a high proportion of cases shared space and time on a ward. As a consequence, 366 (75%) of CDI cases were inpatients (admitted overnight) at the time of sampling, a slightly higher proportion than unsequenced cases (65%, *P *= 0.005). However, 277 (57%) of CDI cases were female and the median age at diagnosis was 79 (interquartile range 67 to 86) years, very similar to unsequenced cases (*P *> 0.6).

### Sample treatment

Stools identified as enzyme immunoassay positive with sufficient sample remaining underwent selective culture. Industrial methylated spirits (0.5 ml) was added to a 0.5 ml fecal sample (pea-sized portion if the stool was formed), and the sample was vortex mixed and incubated at room temperature for 1 h. A loopful was then cultured onto modified Brazier's cycloserine-cefoxitin-egg yolk (CCEY) agar (CCEY agar base containing cycloserine-cefoxitin supplement and 5% defibrinated horse blood), and the plates were incubated anaerobically at 37°C for up to 7 days. A single colony was subcultured onto a Columbia blood agar (CBA) plate and incubated for 48 h, after which colonies giving the characteristic odour and fluorescence under UV illumination were obtained and underwent MLST [11]. For long-term storage, isolates were emulsified in nutrient broth containing 10% glycerol and stored at -80°C.

### DNA preparation

Each previously frozen isolate was inoculated on to a CBA plate and incubated anaerobically for 48 hours at 37°C. A number of colonies were sampled from the resulting growth to be representative of the frozen stock culture, and transferred to a new CBA plate and incubated anaerobically for a further 48 hours at 37°C. DNA was extracted using a commercial kit (FastDNA, MP Biomedicals, Santa Ana, CA, USA; or QIAamp, Qiagen, Hilden, Germany).

### Library preparation and sequencing

DNA was sequenced at the Wellcome Trust Centre for Human Genetics (WTCHG), Oxford, UK, using the Illumina, Inc. (San Diego, CA, USA) sequencing-by-synthesis technology [21,58]. A combination of standard Illumina and in-house protocols were used to produce multiplexed paired-end libraries with an average insert size of approximately 200 bp. Twelve-plex pooled libraries were sequenced on the Genome Analyzer II (GAII) or GAIIx platforms to produce 51 or 100-108 bp reads, respectively, and 96-plex pooled libraries were sequenced on the HiSeq2000 platform to produce 99 or 100 bp paired reads. In some cases, the same pooled libraries were sequenced in two or more lanes or in different runs, then combined to produce a single set of reads for analysis.

### Assembly and variant calling

The full set of properly paired reads from each isolate was mapped to a reference genome using Stampy [22] v1.0.11 without Burrows-Wheeler Alignment [59] pre-mapping, using an expected divergence (substitution rate) of 0.01 and with default values for other program options, to produce BAM files used in subsequent base-calling. All isolates were mapped against the single bacterial chromosome of *C. difficile *strain CD630 [16] (GenBank AM180355.1, GI 115249003, length of 4,290,252 bp). The median depth of coverage (number of reads mapped to reference positions) across all genomes was 39× with an interquartile interval ranging from 28× to 67×. We called single nucleotide variants using the SAMtools [60] 'mpileup' command and with options '-M0 -Q30 -q30 -o40 -e20 -h100 -m2 -D -S'. We only used the single nucleotide variants that met the following criteria: (1) at least 5 reads with at least 1 read in each direction; (2) no other variant within 12 bases; (3) depth of high-quality coverage between the 2.5 and 97.5 percentiles of all sites for that isolate; (4) sites in unique regions of the reference genome, as judged by constructing a mask of all regions with self similarity using BLAST [61]; (5) at least 75% of reads support the call; and (6) a call must be homozygous under a diploid model. This resulted in calls for mean 85% of the positions of the CD630 reference genome.

### Molecular clock estimate from longitudinal data

For the 91 pairs of genomes sequentially isolated from the same patient and described above, we counted the number A of sites called in both genomes, and the number B of these that were called differently (Additional file 1). In line with Figure 2, we need to estimate the mutation rate within a lineage while allowing for the possibility that variable sites may also represent within-host evolution prior to the initial date of sampling. Accordingly, we assumed a model for the data in which B is Poisson distributed with compound parameter p*A+µ*T*A, where p is the instantaneous within-host diversity, µ is the molecular clock rate and T is the time separating the two genomes [24,62]. To estimate the two unknown parameters µ and p in this model, Bayesian inference was performed using the uninformative prior uniform over (0,∞)). The point estimate for the molecular clock rate µ was 3.2 × 10^-7 ^mutations per site per year, with a 95% credibility interval ranging from 1.3 × 10^-7 ^to 5.3 × 10^-7^. The instantaneous within-host diversity p had a point estimate of 5.7 × 10^-8 ^differences per site, with a 95% credibility interval ranging from 1.5 × 10^-8 ^to 11.1 × 10^-8^. This value of p is equivalent to an expected number of differences across the genome in the interval range of 0.06 to 0.48. To check that this estimate is realistic, we fully sequenced the genomes for between 9 and 12 different *C. difficile *colonies grown from individual clinical samples in 7 different experiments (Additional file 6). We found that the diversity between these genomes was between p = 0 and p = 0.47. These results are highly consistent with our estimate above of the instantaneous within-host diversity of *C. difficile *from longitudinal data.

### Comparative analysis of genomes from distinct cases

A global phylogenetic tree was computed using UPGMA [63] on a single genome from each of the 486 CDI cases (Figure 1a). Fifteen groups of closely related genomes were analyzed further as highlighted in Figure 1a. UPGMA is a crude method to compute a phylogeny, but here it was only used to investigate the relationships between STs and to define groups of related genomes within which more precise phylogenetic inference was performed using ClonalFrame [35] (see below). ClonalFrame could not be applied directly to the whole set of 486 genomes because of its high computational cost when applied to many highly diverse genomes. Furthermore, applying ClonalFrame separately to each group allowed us to uncover important evolutionary differences between them.

ClonalFrame [35] version 1.2 was applied to each group separately in order to infer a within-group genealogy accounting for the possible occurrence of homologous recombination. The ClonalFrame model incorporates both mutation and recombination, and is able to disentangle the effects of these two processes on the genetic data. The signal indicating recombination imports are nucleotide differences that are clustered within a genomic interval. Other available software packages for phylogenetic reconstruction do not account for recombination and in this situation could therefore over-estimate branch lengths. The Monte-Carlo Markov Chain [64] (MCMC) within ClonalFrame was run on each group for 20,000 iterations, with the first half discarded as burn-in. Repeat runs with different initial values were performed and compared to confirm convergence and mixing of the MCMC. Since ClonalFrame identifies which substitutions were introduced by mutation (rather than recombination), these can be used in combination with the mutation rate µ estimated from longitudinal data (with the uncertainty being carried through) and the known times of isolation of the genomes in order to infer the mean and 95% credibility intervals of the ages of the common ancestors [32,46,65]. Estimating times back to common ancestors in a phylogenetic context allows for more accurate dating than if sequences were considered in a pairwise fashion, because the age of the common ancestor of a pair is informed not only by the number of differences between this pair but also by their relationship with all other sequences.

The mean age of ancestral nodes are shown by the position on the x-axis in Figure 1b and Figure 0A dditional file 3, and the credibility intervals are shown by the blue bars around them. These figures were drawn using FigTree version 1.3.1 [66]. They represent majority-rule consensus trees based on posterior samples of phylogenies [67]. To assess the plausibility of direct transmission between two cases, the lower bound of the 95% credibility interval around the age of their most recent common ancestor node was used. If the node corresponding to the most recent common ancestor was unresolved in the majority-rule consensus tree, the phylogeny most likely to be compatible with transmission (that is, with the two branches supporting the two cases most closely related) was used in order to be conservative in ruling-out transmission.

### Epidemiological links between patients

In order to validate our genomic analysis of transmission, we compared it with the results of a previous epidemiological study on the same patients [12]. Epidemiological links were made when two CDI cases shared a ST and time on a ward, either (i) after the sample of first case (the 'donor') and before the sample of the second case (the 'recipient') or (ii) before both samples were taken. For each link, the 'minimum infectious period' necessary for transmission to have occurred was defined as the time between the first sample from the potential donor and ward contact with the recipient. The 'incubation period' was defined as the time between this ward contact and the first sample in the recipient. Incubation periods were assumed to be no greater than 12 weeks, and infectious periods no greater than 8 weeks. The subset of 486 CDI cases studied here with whole genome sequencing were considered epidemiologically linked if they were part of a potential transmission network defined by these links; that is, were counted as epidemiologically linked even if there were intermediate linked CDI cases not whole genome sequenced.

### Data availability

The genomic data have been deposited in the NCBI Short Read Archive under accession number 'ERP001520' and can be accessed online [68].

## Abbreviations

CBA: Columbia blood agar; CDI: *Clostridium difficile *infection; MLST: multilocus sequence typing; OUH: Oxford University Hospitals; PCR: polymerase chain reaction; ST: sequence type; TMRCA: time to the most recent common ancestor.

## Competing interests

The authors declare that they have no competing interests.

## Authors' contributions

XD, MW, ASW, DWC, TEAP and RMH conceived the study. DG, AV, LOC, PP and KED performed laboratory work. XD, MC, CLCI, TG, EMB, DJW, RB and PJD contributed to the bioinformatic assembly pipeline. XD, DWE, MC, MAA, ASW, TEAP and RMH analyzed the data. XD, DWE, ASW, DWC, TEAP and RMH wrote the paper. All authors read and approved the final manuscript.

## Supplementary Material

Additional file 1 **Table summarizing the longitudinal data**. Each row corresponds to one of the 91 patients for which two samples were taken on different dates, and the columns indicate when the two samples were taken and how they differed. Click here for file

Additional file 2 **Table summarizing the transmission data**. Each row corresponds to one of the 486 CDI cases described in the main text. Click here for file

Additional file 3 **Figure showing the microevolutionary analysis for all 15 groups**. Equivalent plot to Figure 1b for the 15 groups highlighted in Figure 1a. Click here for file

Additional file 4 **Table comparing the effect of considering pairs within one or three months of each other**. Proportion of pairs of cases for which the TMRCA could be less than six months ago, when considering pairs of cases from the same ST and separated by a maximum of one month (left) or three months (right). Click here for file

Additional file 5 **Figure assessing the strength of agreement of the epidemiological and genomic analyses of transmission**. The arrow at Kappa = 0.49 indicates Cohen's Kappa coefficient of agreement between links produced by the genomic (TMRCA <6 months) and epidemiological (shared time and space on hospital wards) analysis. The histogram shows the density of Kappa arising by chance, estimated using 10,000 random permutations of the epidemiological labels within each ST. Click here for file

Additional file 6 **Table summarizing the results of instantaneous within-host genomic diversity**. Each row corresponds to one of seven experiments where 9 to 12 genomes were sequenced from multiple colonies grown from a single clinical sample. The genetic relationships between the genomes is described, and the average pairwise distance between genomes p is reported in the last column. Click here for file
