# Supplementary material for: Microevolutionary analysis of Clostridium difficile genomes to investigate transmission
Source: Genome Biol. 2012 Dec 21;13(12):R118. doi: 10.1186/gb-2012-13-12-r118 (PMC4056369; doi:10.1186/gb-2012-13-12-r118)
Supplement: Additional file 1 — Table summarizing the longitudinal data. Each row corresponds to one of the 91 patients for which two samples were taken on different dates, and the columns indicate when the two samples were taken and how they differed. [file gb-2012-13-12-r118-S1.PDF]

ST	DATE1	GENOMEID1	DATA2	GENOMEID2	DATE_DIFF	SNPs	SitesCalled
1	30/07/2007	3016	09/01/2008	3036	163	1	3395330
42	16/09/2008	2805	22/10/2008	513	36	1	3438785
18	12/02/2007	3006	30/05/2007	3012	107	1	3341757
1	04/05/2007	3011	25/01/2008	3038	266	2	3354902
1	30/12/2007	2928	26/04/2008	2929	118	0	3358711
1	05/07/2007	1639	24/12/2007	3033	172	0	3308219
1	06/03/2007	2907	14/05/2007	2914	69	0	3364094
17	17/06/2009	499	24/08/2009	518	68	0	3447867
42	07/07/2009	2819	07/04/2010	2860	274	1	3388675
58	24/01/2010	2488	04/06/2010	2880	131	0	3436372
6	19/08/2009	2823	28/09/2009	2831	40	1	3427822
8	08/10/2007	3027	28/02/2008	3039	143	0	3377355
37	25/11/2008	1484	26/11/2008	1485	1	0	3444381
1	03/08/2007	2918	17/09/2007	2923	45	0	3258540
6	31/08/2009	2826	04/11/2009	2835	65	0	3459271
1	16/01/2009	2949	07/05/2009	2998	111	0	3292139
10	11/11/2009	2738	12/11/2009	1574	1	0	3388675
37	27/05/2009	2990	06/07/2009	1493	40	0	3409093
2	18/04/2007	21	08/11/2007	22	204	2	3465037
2	08/07/2008	2933	28/09/2008	2935	82	1	3447453
1	29/01/2007	2906	03/05/2007	2913	94	0	3145237
35	28/04/2009	1580	21/07/2009	1587	84	1	3511645
6	19/09/2007	2742	09/11/2007	2496	51	0	3443151
1	15/11/2008	130	26/05/2009	127	192	0	3427059
1	19/01/2007	3003	12/03/2008	2793	418	1	3342441
42	28/03/2007	1544	18/09/2007	175	174	0	3447895
11	17/11/2009	2838	30/12/2009	2984	43	2	3092993
42	14/09/2007	49	18/09/2007	50	4	0	3434134
42	02/05/2007	2912	26/07/2007	2917	85	0	3266035
42	17/11/2007	2924	20/12/2007	2760	33	0	3370137
8	21/12/2007	3031	10/02/2009	3043	417	3	3178188
44	30/04/2008	2783	02/06/2008	1628	33	0	3396016
1	13/04/2007	44	02/03/2008	3040	324	0	3422264
1	13/01/2009	2948	05/03/2009	2995	51	0	3290780
6	30/01/2007	1543	23/05/2007	35	113	0	3000493
14	03/02/2009	2731	04/02/2009	1461	1	1	3472180
2	16/11/2008	2943	18/02/2009	2950	94	3	3395882
1	08/01/2007	3001	23/04/2007	3010	105	0	3314630
37	28/12/2008	1487	03/01/2009	1488	6	0	3442865
2	11/01/2009	27	05/08/2009	18	206	0	3481184
14	07/08/2008	158	03/10/2008	150	57	1	3476228
36	21/05/2009	2958	15/08/2009	2969	86	0	3272756
3	15/05/2007	2915	28/08/2007	2919	105	1	3402815
1	19/11/2008	1542	06/03/2009	126	107	0	3401562
1	01/03/2007	3007	18/09/2007	3024	201	2	3352764
1	20/08/2007	3018	17/01/2008	3037	150	0	3225983
42	23/09/2007	51	20/10/2007	40	27	0	3441342
6	28/12/2007	2499	02/03/2008	2503	65	0	3439886
63	01/06/2009	2483	07/07/2009	2485	36	0	3556389
17	03/12/2007	218	04/12/2007	219	1	0	3417384
1	05/05/2008	2930	01/07/2008	2932	57	0	3379134
1	03/07/2007	3014	30/10/2007	3028	119	4	3412663
2	22/08/2009	2971	18/11/2009	2979	88	0	3339754
1	13/11/2008	2941	12/01/2009	2992	60	0	3365935
1	15/01/2007	3002	07/04/2007	3008	82	1	3372965
1	28/01/2007	3004	11/08/2008	3041	561	0	3320749
44	26/10/2008	2728	08/12/2008	1597	43	0	3429684
58	11/01/2008	178	09/05/2008	183	119	1	3470807
45	28/10/2008	3060	15/12/2008	3063	48	1	2943156
17	12/08/2007	2721	08/09/2007	497	27	0	3485697
1	30/06/2007	166	16/10/2007	161	108	2	3421727
55	02/11/2009	3073	12/01/2010	3080	71	0	3291756
10	27/02/2009	1551	28/02/2009	1552	1	0	3423653
46	12/08/2007	3017	20/11/2007	3030	100	1	3231109
45	10/08/2009	2968	14/09/2009	2976	35	0	3343648
66	09/09/2009	2974	22/10/2009	2977	43	0	3260955
1	08/06/2007	94	29/09/2007	3026	113	0	3372816
3	08/03/2009	2955	10/05/2009	2957	63	1	3398676
1	18/08/2008	128	19/08/2008	1498	1	0	3405276
42	11/03/2008	2777	02/05/2008	2787	52	0	3441661
10	02/03/2008	2772	03/05/2008	2788	62	0	3429370
44	01/04/2009	1601	14/06/2009	1604	74	0	3443127
10	26/10/2009	1565	27/10/2009	1568	1	0	3399538
4	19/03/2008	39	30/09/2008	2937	195	0	2902990
63	08/06/2009	2484	17/07/2009	2487	39	2	3513811
42	21/11/2007	2757	01/01/2008	2762	41	1	3428043
3	21/12/2009	2994	13/02/2010	2999	54	0	3336802
10	04/08/2007	74	05/08/2007	1640	1	1	3426088
44	11/09/2009	1627	05/10/2009	1611	24	0	3461659
17	02/07/2009	2818	16/08/2009	2822	45	0	3405923
12	05/09/2008	491	06/09/2008	489	1	0	3407467
1	02/08/2007	202	05/09/2007	2921	34	0	3397014
1	04/06/2008	494	05/06/2008	1634	1	0	3248769
17	05/05/2009	504	13/11/2009	2837	192	0	3387552
42	22/10/2007	2746	24/12/2007	2761	63	0	3398093
14	12/06/2009	2960	28/08/2009	2972	77	0	3373574
17	11/09/2007	109	30/04/2008	145	232	2	3451133
35	13/06/2009	1586	14/08/2009	1588	62	0	3520752
1	31/01/2008	154	23/02/2008	155	23	0	3442217
54	02/10/2006	567	15/02/2007	531	136	0	3619348
1	26/09/2008	80	22/01/2009	83	118	0	3397240
