# Supplementary material for: Microevolutionary analysis of Clostridium difficile genomes to investigate transmission
Source: Genome Biol. 2012 Dec 21;13(12):R118. doi: 10.1186/gb-2012-13-12-r118 (PMC4056369; doi:10.1186/gb-2012-13-12-r118)
Supplement: Additional file 2 — Table summarizing the transmission data. Each row corresponds to one of the 486 CDI cases described in the main text. [file gb-2012-13-12-r118-S2.PDF]

ST	ID	DATE
1	205	31/12/2006
1	3001	08/01/2007
1	3002	15/01/2007
1	3003	19/01/2007
1	66	21/01/2007
1	90	28/01/2007
1	3004	28/01/2007
1	2906	29/01/2007
1	206	02/02/2007
1	91	09/02/2007
1	207	12/02/2007
1	3007	01/03/2007
1	67	04/03/2007
1	208	04/03/2007
1	2907	06/03/2007
1	209	10/03/2007
1	68	18/03/2007
1	210	27/03/2007
1	69	28/03/2007
1	70	29/03/2007
1	211	06/04/2007
1	44	13/04/2007
1	45	19/04/2007
1	3011	04/05/2007
1	212	12/05/2007
1	94	08/06/2007
1	95	18/06/2007
1	166	30/06/2007
1	3014	03/07/2007
1	1639	05/07/2007
1	162	14/07/2007
1	163	15/07/2007
1	73	30/07/2007
1	3016	30/07/2007
1	202	02/08/2007
1	2918	03/08/2007
1	204	05/08/2007
1	47	14/08/2007
1	3018	20/08/2007
1	54	01/09/2007
1	55	04/09/2007
1	64	07/09/2007
1	56	05/10/2007
1	87	21/10/2007
1	88	23/10/2007
1	65	10/11/2007
1	216	26/11/2007
1	42	02/12/2007
1	515	14/12/2007
1	221	19/12/2007
1	2928	30/12/2007
1	152	04/01/2008
1	153	18/01/2008
1	154	31/01/2008
1	1469	24/02/2008
1	52	04/04/2008
1	198	15/04/2008
1	514	02/05/2008
1	2930	05/05/2008
1	494	04/06/2008
1	128	18/08/2008
1	199	14/09/2008
1	79	22/09/2008
1	200	24/09/2008
1	80	26/09/2008
1	2941	13/11/2008
1	130	15/11/2008
1	1542	19/11/2008
1	135	24/11/2008
1	1460	28/11/2008
1	151	06/12/2008
1	136	19/12/2008
1	2948	13/01/2009
1	2949	16/01/2009
1	84	31/01/2009
1	85	09/02/2009
1	86	15/02/2009
1	75	25/02/2009
1	3044	10/06/2009
1	1478	18/06/2009
1	2737	20/09/2009
10	74	04/08/2007
10	1641	10/09/2007
10	110	11/09/2007
10	2756	16/11/2007
10	2803	22/12/2007
10	2767	24/01/2008
10	2770	21/02/2008
10	2772	02/03/2008
10	2776	06/03/2008
10	2784	26/04/2008
10	2785	30/04/2008
10	215	30/07/2008
10	1548	14/09/2008
10	1621	26/09/2008
10	1622	15/10/2008
10	1549	21/11/2008
10	1551	27/02/2009
10	1555	05/07/2009
10	1556	22/07/2009
10	1557	13/08/2009
10	1560	17/08/2009
10	1562	22/08/2009
10	1563	28/08/2009
10	1564	14/09/2009
10	1567	27/10/2009
10	1569	27/10/2009
10	1573	29/10/2009
10	2738	11/11/2009
10	1575	07/12/2009
10	1576	13/12/2009
10	1577	23/12/2009
10	1578	29/12/2009
10	1579	29/03/2010
10	2862	11/04/2010
10	2875	27/05/2010
10	2876	27/05/2010
11	1540	20/09/2006
11	1494	25/04/2007
11	2988	11/08/2007
11	2739	04/09/2007
11	2748	24/10/2007
11	2759	18/12/2007
11	2763	04/01/2008
11	1514	14/04/2008
11	2782	28/04/2008
11	2901	07/08/2008
11	2794	13/08/2008
11	1516	08/10/2008
11	2809	15/10/2008
11	2732	17/03/2009
11	1517	16/08/2009
11	2827	02/09/2009
11	1518	05/11/2009
11	2838	17/11/2009
11	1519	03/01/2010
11	2845	04/01/2010
11	2849	27/01/2010
11	2850	28/01/2010
11	1520	31/01/2010
11	2896	01/03/2010
11	1521	03/03/2010
11	2859	06/04/2010
11	2861	08/04/2010
11	2863	14/04/2010
11	2864	17/04/2010
11	2870	09/05/2010
11	2874	26/05/2010
12	2724	02/06/2008
12	562	30/07/2008
12	2725	01/09/2008
12	491	05/09/2008
12	493	12/09/2008
12	488	20/10/2008
12	490	16/11/2008
12	492	19/10/2009
13	149	30/09/2008
13	511	20/08/2009
14	1471	11/02/2008
14	223	28/03/2008
14	158	07/08/2008
14	564	19/08/2008
14	160	26/08/2008
14	2731	03/02/2009
14	2960	12/06/2009
16	194	24/02/2009
17	557	01/11/2006
17	2721	12/08/2007
17	109	11/09/2007
17	1635	12/09/2007
17	2900	12/09/2007
17	503	08/10/2007
17	218	03/12/2007
17	505	26/03/2008
17	102	10/04/2008
17	143	14/04/2008
17	502	10/07/2008
17	148	09/11/2008
17	137	17/11/2008
17	504	05/05/2009
17	508	04/06/2009
17	506	17/06/2009
17	2818	02/07/2009
17	2820	16/07/2009
17	2881	14/06/2010
18	3006	12/02/2007
2	21	18/04/2007
2	98	25/09/2007
2	217	10/11/2007
2	1474	22/05/2008
2	1475	08/07/2008
2	2933	08/07/2008
2	1476	11/08/2008
2	1466	13/08/2008
2	1477	18/08/2008
2	2934	25/08/2008
2	81	07/10/2008
2	1535	17/10/2008
2	475	27/10/2008
2	2943	16/11/2008
2	2945	11/01/2009
2	510	15/05/2009
2	3046	27/07/2009
2	2971	22/08/2009
22	1506	06/08/2007
22	1507	07/08/2007
22	1508	02/04/2008
22	1509	09/08/2008
22	1510	07/06/2009
25	2723	26/04/2008
3	2915	15/05/2007
3	59	16/10/2007
3	2955	08/03/2009
3	566	20/04/2009
3	520	23/04/2009
3	2994	21/12/2009
35	2752	05/11/2007
35	182	08/03/2008
35	2778	13/03/2008
35	1580	28/04/2009
35	1586	13/06/2009
35	1591	24/08/2009
35	1589	19/12/2009
35	1593	05/01/2010
36	2958	21/05/2009
37	1495	17/09/2006
37	553	19/10/2006
37	550	21/10/2006
37	552	30/05/2007
37	1492	26/08/2007
37	2722	04/12/2007
37	89	25/12/2007
37	554	09/08/2008
37	1482	14/09/2008
37	1483	22/10/2008
37	1484	25/11/2008
37	2730	08/12/2008
37	1486	17/12/2008
37	1487	28/12/2008
37	2990	27/05/2009
37	3071	21/09/2009
37	3077	08/12/2009
37	3082	25/01/2010
4	39	19/03/2008
41	224	29/05/2008
41	2515	27/02/2009
41	2440	09/12/2009
42	1536	09/01/2007
42	1544	28/03/2007
42	2912	02/05/2007
42	1465	09/07/2007
42	46	31/07/2007
42	2740	06/09/2007
42	48	10/09/2007
42	49	14/09/2007
42	51	23/09/2007
42	2746	22/10/2007
42	2747	24/10/2007
42	2749	24/10/2007
42	60	26/10/2007
42	2750	31/10/2007
42	2751	05/11/2007
42	2755	10/11/2007
42	2925	17/11/2007
42	2757	21/11/2007
42	2764	10/01/2008
42	2769	31/01/2008
42	2774	04/03/2008
42	2777	11/03/2008
42	2779	24/03/2008
42	2781	16/04/2008
42	1491	07/05/2008
42	2789	17/05/2008
42	2790	06/07/2008
42	2791	10/07/2008
42	2792	22/07/2008
42	2795	18/08/2008
42	2805	16/09/2008
42	2806	17/09/2008
42	2807	17/09/2008
42	2810	10/11/2008
42	2811	22/12/2008
42	2812	19/01/2009
42	2813	04/02/2009
42	2814	30/03/2009
42	2815	15/04/2009
42	2816	27/04/2009
42	2817	28/05/2009
42	2819	07/07/2009
42	2825	27/08/2009
42	2840	26/11/2009
42	2885	22/06/2010
44	43	25/01/2007
44	24	05/07/2007
44	2899	06/07/2007
44	2741	11/09/2007
44	2745	18/10/2007
44	2754	08/11/2007
44	41	14/11/2007
44	2758	05/12/2007
44	2765	17/01/2008
44	560	20/01/2008
44	2766	23/01/2008
44	62	21/02/2008
44	2771	28/02/2008
44	2773	03/03/2008
44	2780	13/04/2008
44	2804	15/04/2008
44	2783	30/04/2008
44	1629	09/06/2008
44	1594	21/06/2008
44	1595	25/06/2008
44	1599	20/08/2008
44	2727	19/09/2008
44	2728	26/10/2008
44	1459	04/11/2008
44	2729	28/11/2008
44	1598	10/03/2009
44	1626	18/03/2009
44	1601	01/04/2009
44	2733	12/05/2009
44	2734	30/05/2009
44	1605	26/07/2009
44	1606	13/08/2009
44	1607	24/08/2009
44	1627	11/09/2009
44	1610	27/09/2009
44	1612	05/10/2009
44	1613	09/11/2009
44	2842	15/12/2009
44	1614	20/01/2010
44	1615	16/02/2010
44	2852	16/02/2010
44	1616	25/02/2010
44	1617	09/03/2010
44	1618	16/03/2010
44	1619	22/03/2010
44	2898	19/04/2010
44	2866	20/04/2010
44	2873	23/05/2010
44	2877	31/05/2010
44	2882	15/06/2010
44	2883	15/06/2010
45	1637	11/12/2007
45	3055	08/01/2008
45	3058	30/03/2008
45	3060	28/10/2008
45	2968	10/08/2009
45	3074	25/11/2009
45	3079	14/12/2009
46	3017	12/08/2007
49	3052	22/09/2007
49	3053	06/11/2007
49	3064	01/02/2009
49	3066	05/07/2009
49	3067	12/07/2009
49	3068	14/08/2009
49	3072	19/10/2009
49	3075	02/12/2009
5	1539	03/10/2006
5	1501	25/01/2007
5	72	08/07/2007
5	1636	20/06/2008
5	2989	25/09/2008
5	1503	03/05/2009
5	1504	27/09/2009
5	1505	17/02/2010
54	567	02/10/2006
54	528	16/10/2007
54	525	29/11/2007
54	529	14/07/2008
54	526	23/10/2008
54	524	27/10/2008
54	532	24/04/2009
54	522	12/05/2009
54	523	01/06/2009
54	2479	25/03/2010
55	3057	03/03/2008
55	3059	23/10/2008
55	3061	06/11/2008
55	3062	16/11/2008
55	3069	17/09/2009
55	3070	20/09/2009
55	3073	02/11/2009
55	3076	06/12/2009
55	3078	10/12/2009
57	157	08/06/2008
57	82	11/11/2008
58	480	09/11/2006
58	533	21/09/2007
58	509	24/09/2007
58	539	07/12/2007
58	537	04/01/2008
58	178	11/01/2008
58	2473	23/03/2008
58	53	09/04/2008
58	544	04/12/2008
58	535	22/01/2009
58	542	09/02/2009
58	2474	30/07/2009
58	2475	04/08/2009
58	2477	19/08/2009
58	2478	11/10/2009
58	2488	24/01/2010
59	1463	03/05/2009
6	1543	30/01/2007
6	1642	04/03/2007
6	2742	19/09/2007
6	2743	20/09/2007
6	2744	24/09/2007
6	2494	21/10/2007
6	2495	25/10/2007
6	2497	06/12/2007
6	2498	11/12/2007
6	2499	28/12/2007
6	2501	12/01/2008
6	2502	21/02/2008
6	2506	13/03/2008
6	222	15/03/2008
6	2507	17/03/2008
6	2508	07/04/2008
6	2509	28/04/2008
6	2510	06/05/2008
6	156	05/06/2008
6	1464	05/06/2008
6	2512	08/06/2008
6	2513	10/07/2008
6	2514	15/07/2008
6	2712	23/07/2008
6	2796	23/08/2008
6	2518	31/08/2008
6	2519	09/09/2008
6	2520	24/09/2008
6	2521	02/10/2008
6	2808	08/10/2008
6	2522	10/12/2008
6	2523	02/02/2009
6	195	22/04/2009
6	76	15/07/2009
6	2525	22/07/2009
6	2524	23/07/2009
6	2526	01/08/2009
6	2821	13/08/2009
6	2823	19/08/2009
6	2824	25/08/2009
6	2826	31/08/2009
6	2828	09/09/2009
6	2830	18/09/2009
6	2832	07/10/2009
6	2833	14/10/2009
6	2834	24/10/2009
6	2836	11/11/2009
6	2839	21/11/2009
6	2841	07/12/2009
6	2846	07/01/2010
6	2847	16/01/2010
6	2848	16/01/2010
6	2851	11/02/2010
6	2854	28/02/2010
6	2856	03/03/2010
6	2857	11/03/2010
6	2858	06/04/2010
6	2867	20/04/2010
6	2868	26/04/2010
6	2872	19/05/2010
6	2884	22/06/2010
63	2482	24/05/2009
63	2483	01/06/2009
63	2484	08/06/2009
63	2486	13/07/2009
63	2713	26/07/2009
66	2974	09/09/2009
67	2504	05/10/2009
7	561	13/08/2008
77	196	14/07/2009
77	197	21/07/2009
8	3027	08/10/2007
8	3031	21/12/2007
8	142	14/04/2008
8	191	09/01/2009
8	1462	30/03/2009
89	77	07/05/2007
