# Supplementary material for: Microevolutionary analysis of Clostridium difficile genomes to investigate transmission
Source: Genome Biol. 2012 Dec 21;13(12):R118. doi: 10.1186/gb-2012-13-12-r118 (PMC4056369; doi:10.1186/gb-2012-13-12-r118)
Supplement: Additional file 4 — Table comparing the effect of considering pairs within one or three months of each other. Proportion of pairs of cases for which the TMRCA could be less than six months ago, when considering pairs of cases from the same ST and separated by a maximum of one month (left) or three months (right). [file gb-2012-13-12-r118-S4.PDF]

Group	STs	Counts	Pairs within 1 month	TMRCA<6months	Proportion	Pairs within 3 months	TMRCA<6months	Proportion
1	ST11	31	27	2	7%	88	14	16%
2	ST5,22	9,5	1	1	100%	1	1	100%
3	ST37	18	7	7	100%	22	14	64%
4	ST1	81	267	167	63%	706	427	60%
5	ST17	19	17	6	35%	30	11	37%
6	ST10	37	40	5	13%	125	6	5%
7	ST44	52	57	2	4%	193	4	2%
8	ST35	8	2	1	50%	4	3	75%
9	ST54,63	10,5	7	4	57%	15	7	47%
10	ST58	16	8	4	50%	19	6	32%
11	ST12,57	8,2	4	3	75%	16	6	38%
12	ST3	7	2	0	0%	4	1	25%
13	ST6,89	62,1	103	5	5%	328	8	2%
14	ST2,13,49,14	18,2,8,7	17	4	24%	58	14	24%
15	ST42	45	66	23	35%	189	53	28%
	Total		625	234	37%	1798	575	32%
	Total excluding ST1		358	67	19%	1092	148	14%
