# Supplementary figures and images for: Microevolutionary analysis of Clostridium difficile genomes to investigate transmission
Source: Genome Biol. 2012 Dec 21;13(12):R118. doi: 10.1186/gb-2012-13-12-r118 (PMC4056369; doi:10.1186/gb-2012-13-12-r118)

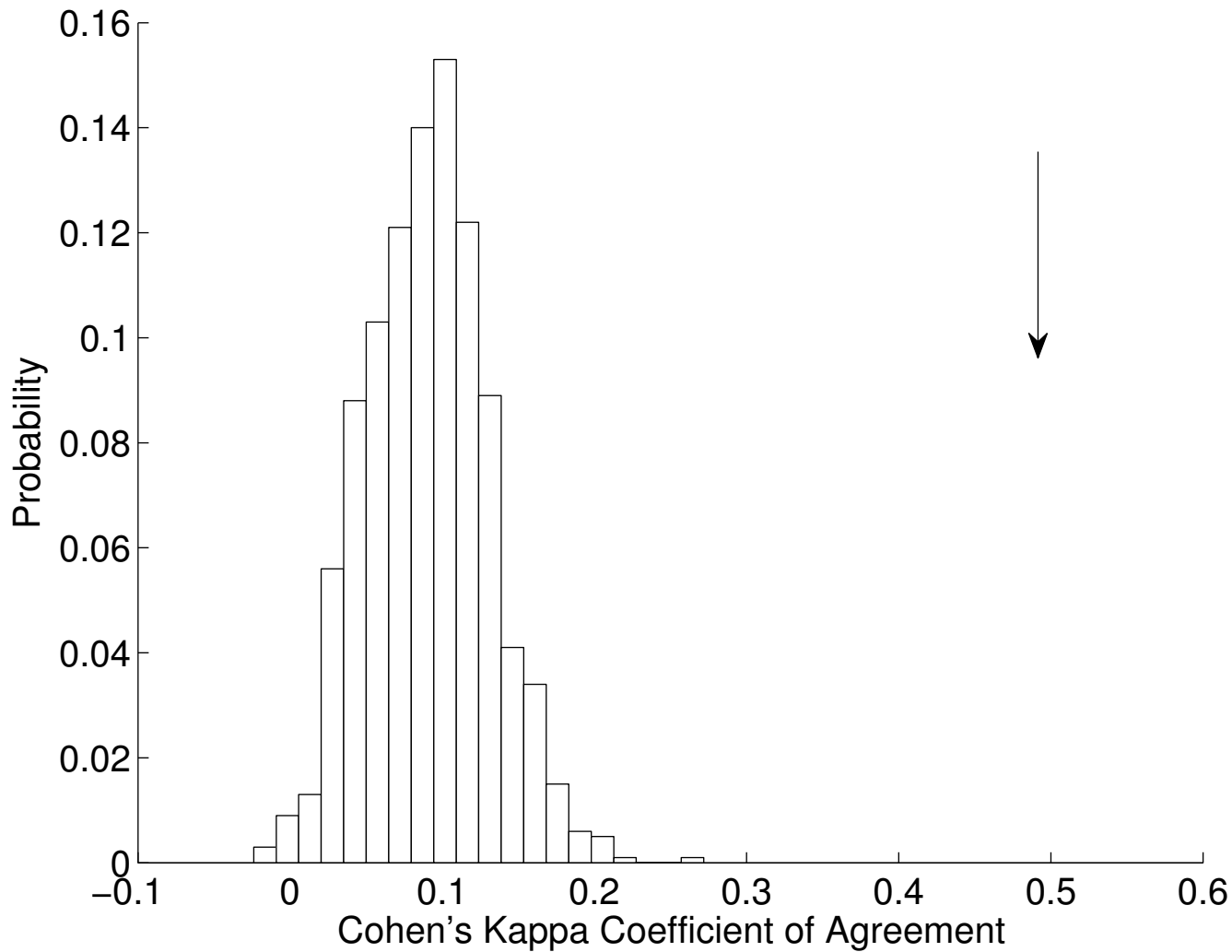

Supplement: Additional file 5 — Figure assessing the strength of agreement of the epidemiological and genomic analyses of transmission. The arrow at Kappa = 0.49 indicates Cohen's Kappa coefficient of agreement between links produced by the genomic (TMRCA <6 months) and epidemiological (shared time and space on hospital wards) analysis. The histogram shows the density of Kappa arising by chance, estimated using 10,000 random permutations of the epidemiological labels within each ST. [file gb-2012-13-12-r118-S5.PDF]
