# Supplementary material for: Microevolutionary analysis of Clostridium difficile genomes to investigate transmission
Source: Genome Biol. 2012 Dec 21;13(12):R118. doi: 10.1186/gb-2012-13-12-r118 (PMC4056369; doi:10.1186/gb-2012-13-12-r118)
Supplement: Additional file 6 — Table summarizing the results of instantaneous within-host genomic diversity. Each row corresponds to one of seven experiments where 9 to 12 genomes were sequenced from multiple colonies grown from a single clinical sample. The genetic relationships between the genomes is described, and the average pairwise distance between genomes p is reported in the last column. [file gb-2012-13-12-r118-S6.PDF]

Experiment	Genomes sequenced	Description of result	Average diversity pi
1	12	all identical	0
2	10	all identical	0
3	12	all identical	0
4	12	11 identical, 1 with 1 SNP	0.17
5	9	7 identical, 2 with same 1 SNP	0.39
6	10	7 identical, 3 with same 1 SNP	0.47
7	9	all identical	0
