# Supplementary material for: Microevolutionary analysis of Clostridium difficile genomes to investigate transmission
Source: Genome Biol. 2012 Dec 21;13(12):R118. doi: 10.1186/gb-2012-13-12-r118 (PMC4056369; doi:10.1186/gb-2012-13-12-r118)

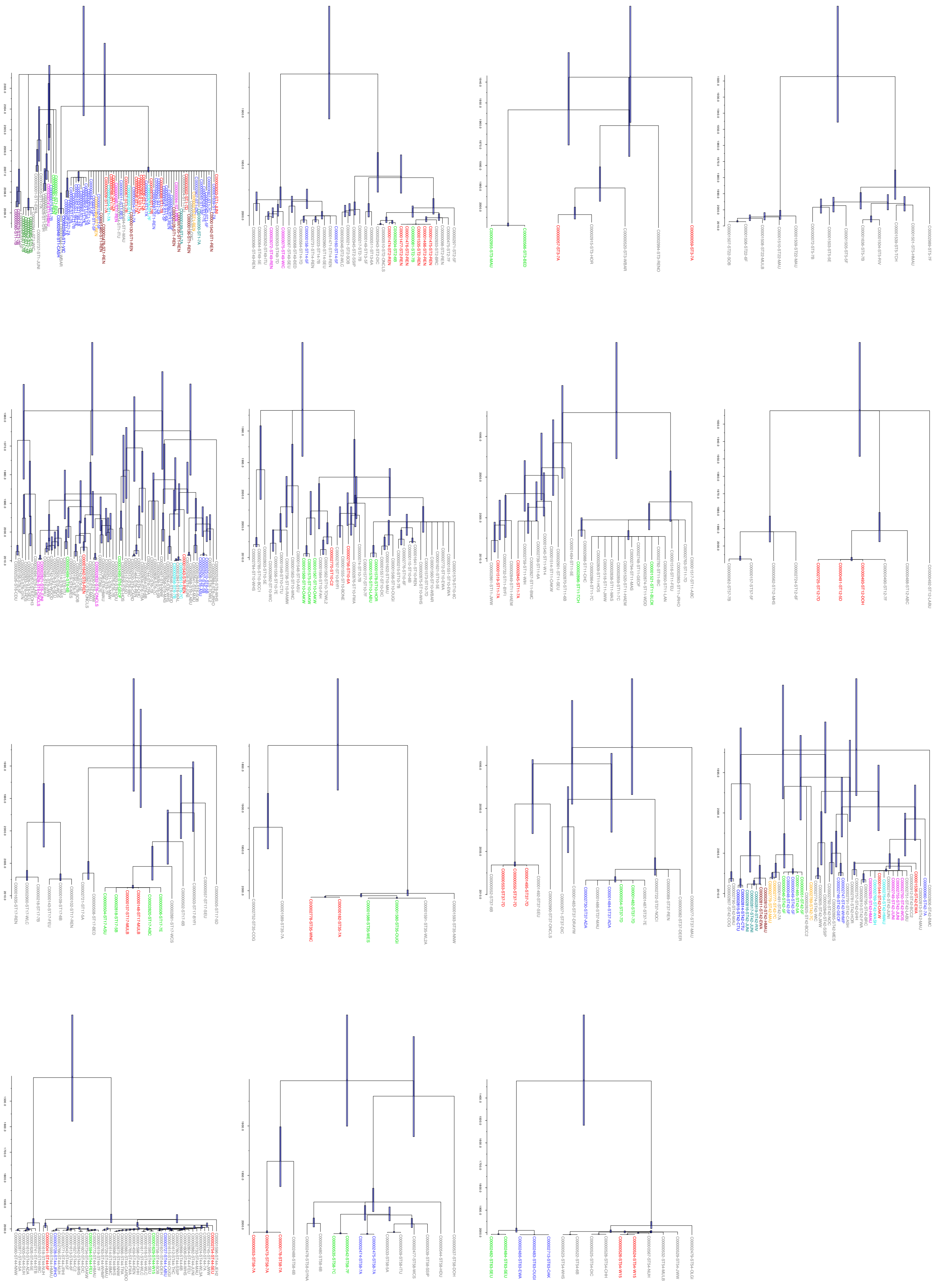

Supplement: Additional file 3 — Figure showing the microevolutionary analysis for all 15 groups. Equivalent plot to Figure 1b for the 15 groups highlighted in Figure 1a. [file gb-2012-13-12-r118-S3.PDF]
